# Diurnal Cortisol Profiles and Veteran Status

**DOI:** 10.1093/geroni/igaa057.2182

**Published:** 2020-12-16

**Authors:** Jennifer Piazza, Scott Landes, Natalia Dmitrieva

**Affiliations:** 1 California State University, Fullerton, Fullerton, California, United States; 2 Aging Studies Institute – Syracuse University, Syracuse, New York, United States; 3 Northern Arizona University, Flagstaff, Arizona, United States

## Abstract

Although veterans typically have better health and lower mortality risk than nonveterans earlier in life, a crossover occurs in later life, resulting in comparatively poorer health and higher mortality risk in older age. Alterations in biomarkers of physical health could be an initial indicator of this crossover. The current study uses data from the second wave of the Midlife Development in the United States (MIDUS) survey. Previously validated cortisol profiles (Dmitrieva et al. 2013) were used to categorize participants (N = 1101, age range: 33-88) into normative and non-normative groups, that were examined in relation to veteran status. Results revealed a significant age by veteran interaction (β = .0331, p = .038), indicating that older veterans were less likely to belong to the normative profile group than non-veterans and younger veterans. Results demonstrate the need to explore underlying physiological mechanisms linking military service with poorer health in later life. Part of a symposium sponsored by the Aging Veterans: Effects of Military Service across the Life Course Interest Group.

